# Mapping of fiber quality QTLs reveals useful variation and footprints of cotton domestication using introgression lines

**DOI:** 10.1038/srep31954

**Published:** 2016-08-23

**Authors:** Shu-Wen Zhang, Xie-Fei Zhu, Liu-Chun Feng, Xiang Gao, Biao Yang, Tian-Zhen Zhang, Bao-Liang Zhou

**Affiliations:** 1 State Key Laboratory of Crop Genetics & Germplasm Enhancement, Nanjing Agricultural University, Nanjing 210095, China

## Abstract

Fiber quality improvement is a driving force for further cotton domestication and breeding. Here, QTLs for fiber quality were mapped in 115 introgression lines (ILs) first developed from two intraspecific populations of cultivated and feral cotton landraces. A total of 60 QTLs were found, which explained 2.03–16.85% of the phenotypic variance found in fiber quality traits. A total of 36 markers were associated with five fiber traits, 33 of which were found to be associated with QTLs in multiple environments. In addition, nine pairs of common QTLs were identified; namely, one pair of QTLs for fiber elongation, three pairs for fiber length, three pairs for fiber strength and two pairs for micronaire (qMICs). All common QTLs had additive effects in the same direction in both IL populations. We also found five QTL clusters, allowing cotton breeders to focus their efforts on regions of QTLs with the highest percentages of phenotypic variance. Our results also reveal footprints of domestication; for example, fourteen QTLs with positive effects were found to have remained in modern cultivars during domestication, and two negative qMICs that had never been reported before were found, suggesting that the qMICs regions may be eliminated during artificial selection.

Cotton (*Gossypium* spp.) is cultivated worldwide for its fiber, and improving fiber quality has been a global goal for cotton breeders. Cotton, as a highly important industrial crop, grows in more than 80 countries located in tropical and subtropical regions^1^. The genus *Gossypium* comprises approximately 45 diploid species and five tetraploid species. *G. hirsutum*, *G. barbadense*, *G. herbaceum* and *G. arboreum* have been extensively cultivated around the world. *G. hirsutum* makes up >90% of the total global cotton production and is generally referred to as Upland cotton^2^. However, numerous studies have shown that Upland cotton has a low level of genetic diversity, which has further decreased in the past century due to the extensive artificial selection in limited cotton resources and the overuse of relatively few cultivars with high yields in larger areas for both breeding and production purposes^3^. Feral Upland cotton not only has the extensive adaptability and high resistance to biotic and abiotic stress of wild types, but also has characteristics of cultivars due to a lack of artificial selection and human domestication, and is therefore a valuable resource for breeding. In addition, it can also be used for revealing the footprints of domestication. The *G. hirsutum* races richmondii acc. TX-256 and *yucatanense* acc. TX-1046 are two feral landraces of Upland cotton that are propitious to improving fiber quality of cultivars and should therefore be introduced into upland cotton to widen its genetic base^4^. Fibers of commercial *Gossypium* species (such as *G. hirsutum* TM-1) are very elongated and thickened seed epidermal cells that may be spun into yarn. Primitive or feral landrace cottons, such as *G. hirsutum* race *yucatanense*, produce shorter fibers, which, though not spinnable, probably represented attractive targets for aboriginal domesticators^5^. Starting from a rangy, perennial shrub with a poorly synchronized fruit set, low yield, photoperiod sensitivity and small seeds that required scarification for germination *in vitro*, human selection over 5 millennia has transformed *G. hirsutum* into a high yield, annualized row-crop with a heavy fruit set, photoperiod insensitivity and seeds that germinate readily upon planting^6^. Domestication is an evolutionary process through which plants and animals change at the genetic level, becoming morphologically and physiologically different from their wild ancestors^7^. Such divergence is the result of a combination of artificial selection and other evolutionary forces determined by the manipulation of plants by humans^8^.

The cotton industry uses fiber properties determined by automated high-volume instruments (HVIs) as parameters for predicting yarn quality and selecting the right raw cotton materials to produce different qualities of yarns^9^. Physical properties, such as length, strength, maturity (degree of thickness), and fineness, determine the value and quality of cotton fibers and the yarn spun from them. Fiber quality is a complex quantitative trait that is a composite of many other traits, such as fiber length (FL), Fiber uniformity (FU), fiber strength (FS), fiber elongation (FE) and micronaire (MIC). Each fiber quality trait is influenced by many genes, and each gene has variable effects on fiber quality^10^. These traits are also affected by biotic stressors, including premature senescence, *Verticillium* wilt, insects and weeds, and by abiotic stress conditions, including flooding, extreme temperatures and drought^11^,^12^. Researchers have detected QTLs associated with fiber properties from both intraspecific populations of *G. hirsutum* and interspecific crosses between *G. hirsutum* and *G. barbadense*, as well as their later generations^13^,^14^,^15^,^16^,^17^. At the same time, the large number of differentially expressed genes highlights the complex alteration of global gene expression machinery that resulted from human selection of a longer, stronger, and finer fiber. Several previous reports have focused on the development of fiber cells and proteomic analyses to detail the evolutionary consequences of strong directional selection for enhanced fiber traits^6^,^18^,^19^,^20^,^21^. Here, we present a detailed QTL analysis of five fiber quality traits to reveal footprints of domestication.

Most genetic maps of cotton species that are used as a basis for research were constructed from interspecific populations. Numerous genetic linkage maps also have been constructed from interspecific populations (*G. barbadense* × *G. hirsutum*) and are used in QTL mapping^22^,^23^,^24^,^25^. While genetic maps of interspecific populations are already plentiful, many genetic maps used for QTL mapping have also been constructed from intraspecific Upland cotton populations^16^,^26^,^27^,^28^,^29^. A high-density genetic linkage map would be greatly beneficial to the development of chromosome segment introgression lines (ILs) via marker-assisted selection (MAS). In cotton, several IL populations have been developed using *G. barbadense* as a donor parent and *G. hirsutum* as a recipient parent (genetic backgrounds) and these IL populations have been used to identify QTLs and genomic segments conferring yield and fiber quality traits^30^,^31^,^32^,^33^. ILs are therefore ideal for genome research and for MAS breeding programs, which have become important tools for permitting the characterization of bidirectional target regions. In our previous research, we constructed a joint genetic linkage map using two BC_1_ populations of three Upland cotton parents, and developed a total of 115 ILs, where TM-1, the genetic standard of Upland cotton, was used as the common recipient and its two feral landraces, TX-256 and TX-1046, were used as the donors. The total length of the introgression segments of 66 ILs derived from TX-256 was 1104.15 cM, which accounted for 28.40% of the whole introgression fragment. The length of introgression fragments of 49 ILs from TX-1046 was 2783.60, accounting for 71.60% of the whole introgression fragment^17^. These two sets of ILs were first developed from intraspecific populations of cotton.

In this study, the above genetic linkage map and ILs from TX-256 and TX-1046 were used to map QTLs of fiber quality traits, identify common QTLs and QTL clusters, and mine useful variation from feral landraces, contributing to understanding the process of cotton domestication.

## Results

### Fiber quality performance of ILs

Two IL populations were planted in four different environments. Their fiber quality performances are presented in Table 1. In all four environments, the mean values of all traits in IL population derived from TM-1 × TX-256 (Pop1) were higher than those in IL population derived from TM-1 × TX-1046 (Pop2), and the values in Pop1 were closer to that of the recurrent parent TM-1. The co-efficient of variation (CV) values of fiber strength (FS) (8.55–12.85% and 8.24–12.91%) in the two populations were much higher than those of the other traits shown (Table 1) while micronaire (MIC) showed the lowest CV values (3.77–5.96% and 2.50–5.40%). The basic parameters of the five traits in the two populations in the four environments were different, but their trends were fairly consistent. The ILs from each population showed significant differences (*p* = 0.01) in the values of all five fiber traits and all five fiber traits also exhibited significant interactions (*p* = 0.01) of ILs × Environments in the two IL populations (Table 2). As expected, all fiber traits of the ILs displayed a high degree of variability in all environments. FS had the largest ILs × Environments interaction (15.17** and 19.68**, *p* = 0.01). The ILs × Environments interaction was the lowest for MIC (1.74** and 2.26**, *p* = 0.01). As shown in Table 3, positive correlations (*p* = 0.05) between FL and FS, and MIC and FE were found to be statistically significant, whereas as were the negative correlations (*p* = 0.05) between FL and MIC, and FS and MIC in both populations. FE was significantly positively correlated (*p* = 0.01) with FL and FS in E4 and significantly negatively correlated (*p* = 0.01) with FL and FS in the other three environments. In addition, positive correlations (*p* = 0.05) between FS and FU were only found in Pop1.

### Comparative QTL mapping of two IL populations

The positions and percentages of the phenotypic variance explained (PVE) by markers of the QTLs are given in Table 4. A total of 36 markers were associated with the five investigated fiber quality traits, 33 of which were found to be associated with QTLs in multiple environments (Fig. S1). Twenty and sixteen SSR markers existed in the At- and Dt-subgenomes, respectively. In total, 60 QTLs were found, of which 31 and 29 were detected in the At- and Dt-subgenomes, respectively. These QTLs explained 2.03–16.85% of PVE, with an average of 6.26% explained in all five fiber quality traits.

### Fiber length

There were 21 QTLs of fiber length (qFLs) detected, of which ten and eleven QTLs were detected in Pop1 and Pop2, respectively. In Pop1, five qFLs increased the average FL by 0.76 mm (ranging from 0.35 to 1.29 mm); their PVE varied from 4.11% to 12.54%. The remaining five decreased it by 0.71 mm (ranging from 1.81 to 0.23 mm); their PVE varied from 3.47% to 9.43%. Seven and one qFLs could be detected in two and three different environments, respectively. Only two qFLs were detected in all four different environments and could be taken as stable FL QTLs, one (qFL-Pop1-D2-1) decreased the average FL by 0.84 mm (ranging from 0.40 to 1.22 mm) and another (qFL-Pop1-A8-1) increased by 0.52 mm (ranging from 0.35 to 2.06 mm). Their PVE varied from 4.63% to 8.99% and from 4.11% to 8.83%, respectively. Chrs. A2, A8, A11, D1, D2, D5, D6 and D13 were each found to have one qFL, and Chr. D8 had two qFLs.

In Pop2, three qFLs increased the average FL by 1.02 mm (ranging from 0.33 to 2.02 mm); their PVE varied from 3.09% to 11.27%. The remaining eight decreased it by 0.94 mm (ranging from 0.16 to 3.11 mm); their PVE varied from 3.24% to 15.53%. One, three, and four qFLs could be detected in one, two, and three different environments, respectively. The remaining three qFLs, qFL-Pop2-D8-3, qFL-Pop2-A7-1 and qFL-Pop2-A5-1, were detected in four different environments, decreasing the average FL by 0.56 mm (0.33 to 0.98 mm), 0.59 mm (0.33 to 2.35 mm) and 1.58 mm (0.95 to 2.58 mm), respectively. Their PVE varied from 3.67–7.41%, 4.06–15.53% and 4.04–1.04%, respectively. Chrs. A3, A5, A7, A9, D1, D2, and D8 were each found to have one qFL, and Chrs. A8 and D11 each had two qFLs (Table 4).

### Fiber strength

Twelve QTLs of fiber strength (qFSs) were detected in total. There were seven qFSs in Pop1, four of which increased the average FS by 1.04 cN/tex (ranging from 0.22 to 2.72 cN/tex); their PVE varied from 2.70% to 14.47%. The remaining three QTLs decreased it by 1.26 cN/tex (ranging from 0.27 to 3.53 cN/tex); their PVE varied from 2.03% to 5.80%. One and three qFSs were detected in two and three different environments, respectively. Three qFSs were detected in all four different environments and could be taken as stable FS QTLs. Two alleles from feral cotton, qFS-Pop1-D2-1 and qFS-Pop1-A13-1, decreased the average FS by 1.51 cN/tex (ranging from 0.40 to 2.71 cN/tex) and 1.96 cN/tex (ranging from 1.17 to 3.53 cN/tex), respectively; their PVE varied from 2.30% to 5.80% and from 2.03% to 4.91%, respectively. Another (qFS-Pop1-A5-1) increased the average FS by 0.75 cN/tex (ranging from 0.44 to 1.35 cN/tex); its PVE varied from 4.77% to 13.34%. Chrs. A3, A5, A6, A8, A13, D2 and D8 were each found to have one qFS.

There were five qFSs in Pop2, three of which increased the average FS by 1.89 cN/tex (ranging from 0.45 cN/tex to 3.47 cN/tex); their PVE varied from 2.93% to 6.64%. The remaining two QTLs decreased it by 1.14 cN/tex (ranging from 0.26 cN/tex to 3.11 cN/tex); their PVE varied from 2.95% to 5.78%. Three and two qFSs were detected in two and three different environments, respectively. No qFS was detected in all four different environments. Chrs. A8, A13 and D5 were each found to have one qFS, and Chr. D2 had two qFSs (Table 4).

### Micronaire

A total of twelve QTLs of micronaire (qMICs) were detected in the four environments. There were six qMICs detected in Pop1; two increased the average micronaire value by 0.48 (ranging from 0.12 to 0.83); their PVE varied from 4.28% to 14.89%). The remaining four decreased itby 0.37 (ranging from 0.14 to 0.63); their PVE varied from 4.28% to 15.37%. Two and three qMICs were detected in two and three different environments, respectively. Only one qMIC was detected in all four different environments and could be taken as stable MIC QTL, increasing the average MIC value by 0.56 (ranging from 0.39 to 1.68); its PVE varied from 6.17% to 14.89%. Chrs. A2, A3, D8 and D11 were each found to have one qMIC, and Chr. D1 had two qMICs.

There were six qMICs detected in Pop2; two increased the average micronaire value by 0.23 (ranging from 0.11 to 0.61); their PVE varied from 3.24% to 7.27%. The remaining four decreased it by 0.58 (ranging from 0.12 to 0.92); their PVE varied from 3.07% to 8.68%. One, one and two qMICs were detected in one, two, and three different environments, respectively. Two qMICs were detected in all four different environments and could be taken as stable MIC QTLs, one decreasing the average MIC value by 0.56 (ranging from 0.35 to 2.25) and another increasing by 0.18 (ranging from 0.11 to 0.53). Their PVE varied from 3.53% to 8.68% and from 3.14% to 4.82%, respectively. Chrs. A6, A8, D1, D2, D8 and D11 were each found to have one qMIC (Table 4).

### Fiber uniformity

In addition, there were four QTLs of fiber uniformity (qFUs) were detected in the two populations; two of which were detected in Pop1. Two QTLs decreased the average FU value by 1.08% (ranging from 0.66 to 1.29%). The PVE varied from 4.47% to 14.12%. One qFU was detected in one environment and another was detected in three different environments. Chrs. A2 and D6 were each found to have one qFU.

Two qFUs were detected in two environments in Pop2, qFU-Pop2-A7-1 having the same effects (both negative) while qFU-Pop2-A5-1 showing different effects in two different environments. The PVE varied from 3.39% to 9.35%. Chrs. A5 and A7 were found to have one qFU each (Table 4). No qFU was detected in all four different environments in the two populations.

### Fiber elongation

There were eleven QTLs of fiber elongation (qFEs) detected, six of which were detected in Pop1. In Pop1, five QTLs decreased the average FE value by 0.39% (ranging from 0.26 to 0.77%); their PVE varied from 4.11% to 9.53%. The remaining one QTL increased it by 0.49% (ranging from 0.39 to 0.64%), its PVE varied from 6.03% to 16.85%. Chrs. A3, A7, A8, A11, D5 and D13 were each found to have one qFE. Two and four qFEs could be detected in two and three different environments, respectively.

The other five qFEs were detected in Pop2. In Pop2, three QTLs decreased the average FE value by 0.54% (ranging from 0.16 to 1.46%); their PVE varied from 5.18% to 14.33%. The remaining two QTLs increased it by 1.15% (ranging from 0.55 to 1.87%); their PVE varied from 5.68% to 15.51%. Chrs. A5, A7, A9, A13 and D8 were each found to have one qFE. Four and one qFEs could be detected in two and three different environments, respectively (Table 4). No qFE was detected in all four different environments in the two populations.

### QTLs common to both IL populations

QTLs for the same trait detected in two different IL populations were defined as “common” QTLs when their confidence intervals overlapped, since we used TM-1 as one common parent. In our analysis, nine pairs of common QTLs, namely, three pairs of QTLs for FL, three pairs of QTLs for FS, two pairs of QTLs for MIC and one pair of QTLs for FE, which distributed on six different chromosomes, were detected in these two IL populations (Table 5). These nine pairs of QTLs showed an additive effect, in the same direction, in these two IL populations.

Five pairs of common QTLs decreased the phenotypic value of fiber quality traits, therefore these are undesirable QTLs. Two pairs of QTLs for FL, qFL-Pop1-D2-1 and qFL-Pop2-D2-2, and qFL-Pop1-D8-2 and qFL-Pop2-D8-3, decreased the fiber length. Two pairs of QTLs for FS, qFS-Pop1-A13-1 and qFS-Pop2-A13-2, and qFS-Pop1-D2-1 and qFS-Pop2-D2-3, decreased the fiber strength. One pair of QTLs for FE, qFE-Pop1-A7-1 and qFE-Pop2-A7-2, decreased the fiber elongation. qFE-Pop1-A7-1 in Pop1 in E1, E3 and E4 on Chr. A7 at the position of 59.66 cM decreased the fiber elongation; as did qFE-Pop2-A7-2 in Pop2 in E1, E2 and E3 on Chr. A7 at the position of 56.56 cM (Table 5). The results indicate that these five pairs of common QTL anchored regions are still in the untapped and feral condition, and further validate that these chromosome regions in modern cultivars have been domesticated by human selection.

We also detected four pairs of common QTLs, namely, qFL-Pop1-A8-1 and qFL-Pop2-A8-2, qFS-Pop1-A8-1 and qFS-Pop2-A8-2, qMIC-Pop1-D1-2 and qMIC-Pop2-D1-3, and qMIC-Pop1-D8-1 and qMIC-Pop2-D8-2, which are favorable QTLs for improving fiber quality. The QTL qFL-Pop1-A8-1 in Pop1 in E1, E2, E3 and E4 on Chr. A8 at the position of 26.33 cM increased the fiber length. Furthermore, qFL-Pop2-A8-2 (at the position of 22.80 cM) was detected in Pop2 in E2 and also increased fiber length. On Chr. A8 at positions 26.33 cM and 22.80 cM, we anchored one pair of common QTLs (qFS-Pop1-A8-1 and qFS-Pop2-A8-2) that could increase the fiber strength (Table 5). The results indicate that these four common QTLs can be used to improve fiber quality in cotton breeding.

### QTL clusters

A QTL cluster is defined as a densely populated QTL region of the chromosome that contains multiple QTLs associated with various traits^34^. In this study, we found five QTL clusters on five chromosomes (Table 6). QTL clusters allow cotton breeders to focus their efforts on regions with the most QTLs with the highest percentages of phenotypic variance. There were four QTLs in the A7 cluster, where the approximate position was 56.56–59.66 cM. The additive effect of qFE-Pop1-A7-1 detected in Pop1 decreased the phenotypic value, and the additive effects of qFE-Pop2-A7-2, qFL-Pop2-A7-1 and qFU-Pop2-A7-1 from Pop2 decreased the phenotypic value. The A8-cluster contained 6 QTLs. qFL-Pop1-A8-1 and qFS-Pop1-A8-1, which were detected in Pop1, had an additive effect, and qFL-Pop2-A8-2 and qFS-Pop2-A8-2 from Pop2 also had an additive effect. There were five QTLs in the D1-cluster. Three qMICs from two different populations had an additive effect, synchronously. qFL-Pop1-D1-1, which was detected in Pop1, also had an additive effect, while qFL-Pop2-D1-2 from Pop2 had a negative effect. The D2-cluster contained five QTLs. qFL-Pop1-D2-1 and qFS-Pop1-D2-1 from Pop1, and qFL-Pop2-D2-2 and qFS-Pop2-D2-3 from Pop2 had negative effects, synchronously. There were five QTLs in the D8-cluster, whose approximate position was 64.40–64.86 cM. qFL-Pop1-D8-2 and qMIC-Pop1-D8-1 from Pop1, and qFL-Pop2-D8-3 and qMIC-Pop2-D8-2 from Pop2 had negative additive effects (A < 0).

## Discussion

Introgression lines are ideal populations for QTL mapping of complex traits. ILs have the potential to uncover new alleles from feral landraces, develop genome-wide genetic stocks and identify genes for functional genomics research^35^. The introgressed chromosome segments of Pop1 originated from *G. hirsutum* race *richmondii* acc. TX-256, while the introgressed chromosome segments of Pop2 originated from *G. hirsutum* race *yucatanense* acc.TX-1046. In four environments, the mean values of all fiber quality traits in Pop1 were higher than that in Pop2, and the values in Pop1 were closer to that of the recurrent parent TM-1 (Table 1), suggesting that TX-1046 was much more primitive in evolution than TX-256. Myriad semi-domesticated forms and landraces span the wild-to-domesticated continuum^36^, so TX-256 is thought to be one of the semi-domesticated landraces. In Table 4, we compared the QTLs detected in Pop1 and Pop2, and found that the ratio of negative effect QTLs in Pop1 (17/31) was slightly lower than that in Pop2 (17/29), illustrating that some negative QTLs were eliminated in TX-256. The co-efficient of variation (CV) values of FS in the two populations were much higher than those of the other traits shown in Table 1, and the lowest CV was found for MIC. There were significant differences between the ILs × Environments interactions for all five fiber traits (Table 2). As with the CV shown in Table 1, FS had the largest ILs × Environments interaction, and is therefore likely to be more easily affected by the environment than the other traits investigated. The ILs × Environments interaction was the lowest for MIC, suggesting that it is much more stable than the other traits. These conclusions are very similar to those of previous reports^9^,^28^,^37^.

In the present report, a total of 60 QTLs were detected in two IL populations in four different environments. Eighteen markers associated with multiple traits were also observed. Many markers, such as BNL3255c, NAU1085 and TML21, were associated with several traits. Five QTL clusters in this study resembled those of Said *et al*.^13^ (Table 6). The A7 cluster, which had an approximate position of 56.56–59.66 cM, was similar to c7-cluster-Gh × Gb-4:55–79 cM. The A8 cluster, which had an approximate position of 22.80–26.33 cM, was similar to c8-cluster-Gh-2:21–31 cM. The D1 cluster, which had an approximate position of 73.07–96.96 cM, was similar to c15-cluster-Gh-3:49–68 cM. The D2 cluster, which had an approximate position of 70.94–73.80 cM, was similar to c14-cluster-Gh-2:76–91 cM. The D8-cluster, which had an approximate position of 64.40–64.86 cM, was similar to c24-cluster-Gh-2:41–62 cM. These common QTLs, hotspot QTLs and clustered QTLs have high reliability and can be utilized for MAS to improve the fiber quality of Upland cotton.

Domestication was the outcome of a cultural change that involved a different human-nature relationship^38^. Twenty-seven of the QTLs identified in our study had been reported before (Table 7), 14 of which had positive additive effects on fiber quality, while the other 13 QTLs had negative additive effects. In addition, two MIC QTLs had never been reported. Fourteen positive QTLs, including two qFEs, four qFLs, five qFSs and three qMICs, remained during domestication, and these could be rare desirable QTLs in untapped landraces and would be valuable resources for breeding. In contrast, thirteen QTLs (4 qFEs, 7 qFLs, 1 qFU and 2 qMICs) had negative additive effects on fiber quality in these two feral landraces, indicating that these alleles are still in a primitive condition in the feral cotton accessions. For example, qFL-Pop1-D8-2 and qFL-Pop2-D8-3 at positions 64.40 and 64.86 cM on Chr.D8 in the target ILs, IL-D8-5 (FL: 28.77 mm) and IL-D8-6 (FL: 27.96 mm), respectively, decreased the fiber length. The t-test results indicated the FLs of these two IL populations were significantly different to that of TM-1 (30.11 mm). This demonstrates that the introgression fragments from the two feral parents have negative effects on fiber quality, reducing the fiber length and strength. Additionally, two new undesirable MIC QTLs detected in the present study were also introgressed from the two feral landraces; qMIC-Pop1-D11-1 and qMIC-Pop2-D11-2, which were on Chr. D11 at positions 6.75 and 11.58 cM, respectively. This pair of common qMICs were not only absent from the hotspots identified by previous reports, but were also distributed in new regions of the cotton genome, and the additive effects of this one pair of common QTLs were negative, decreasing the fiber quality, suggesting that these regions were eliminated under artificial selection. Therefore, there are a number of QTL regions showing negative effects on fiber quality in feral landraces. Moreover, comparing the fiber quality QTLs detected in more than three environments, we found that the number of QTLs showing negative effects on fiber quality is much more than that of QTLs showing positive effects (20 negative QTLs: 10 positive QTLs). The results demonstrate that during the long-term cultivation of cotton, modern cultivated varieties have been subjected to strict artificial selection and domestication, resulting into a great amount of elimination of originally undesirable alleles and the gradual accumulation of desirable sites, especially those conferring fiber quality traits, resulting in the generation of high quality fibers to meet the needs of the textile industry. These materials lay the foundation for research on the history of cotton domestication. Interestingly, compared the yield-related trait QTLs detected in more than three environments, we also found that the number of QTLs showing negative effects on fiber yield-related traits is much more than that of QTLs showing positive effects (25 negative QTLs:15 positive QTLs) (Supplementary Information Table S1). When comparing our results with those of our previous report^17^, and a report by Said *et al*.^13^ on intraspecific and interspecific populations, for example, we found that seven qBWs, five qLPs and five qSIs had never been reported before. Of these QTLs, five qBWs, two qLPs and two qSIs had negative additive effects that decreased the corresponding yield-related trait of the target ILs, suggesting that these sites were untapped in feral landraces and the regions in question were eliminated during domestication. Meanwhile, two qBWs, three qLPs and three qSIs increased the target IL yield-related traits and were detected in only one or two environments, suggesting that these QTLs were unstable and difficult to select during artificial selection, leading to their reluctant reuse in breeding. Comparative QTL analysis is a powerful tool for unraveling the genetic basis of domestication in plant families^39^.

In conclusion, we mapped 60 QTLs of fiber quality traits in four environments using two IL populations, and identified nine pairs of common stable QTLs that can be further used in MAS for improving fiber quality traits in cultivars. We also identified five QTL clusters. Fourteen QTLs had positive additive effects and remained during domestication, and these could be used for breeding in the future; two qMICs had never been reported before and had negative effects, suggesting that the regions were eliminated during artificial selection. The diversity in these QTLs revealed the footprints of domestication. ILs are ideal populations for QTL mapping of complex traits. We believe that these ILs are useful for the improvement of fiber quality traits, and in genetic research on domestication.

## Materials and Methods

### Plant materials

In our previous study, we constructed a joint genetic linkage map using two BC_1_ populations from three Upland cotton parents. A total of 622 SSRs were identified in the genetic map. The total recombination length of the map was 3594.70 cM and the average distance between the adjacent markers was 5.04 cM^17^. Based on the genetic map, 352 (Pop1) and 677 (Pop2) BC_3_F_2_ generation individuals were used to genotype the introgression lines, respectively. From the BC_3_F_2_ generation, the introgressed segments for each individual were checked again by SSR markers and the lines having redundant segments were removed. And then, ILs were selected according to agronomic traits (such as boll weight and boll shape), morphological traits (such as leaf shape, plant type, petal color and pollen color) and fiber quality traits (such as fiber length, fiber strength and micronaire), which were significantly different from TM-1. Finally, a total of distinctive 115 ILs were developed and evaluated the introgression segments, where TM-1, the genetic standard of Upland cotton, was used as the common recipient and its two feral landraces, TX-256 and TX-1046, were used as the donors. The total length of the introgressed segments spanned 3,887.75 cM, with an average length of 11.15 cM between the segments, and this represented 71.55% of the tetraploid cotton genome. The total length of introgression segments of the 66 ILs derived from TX-256 was 1,104.15 cM, which accounted for 28.40% of the whole introgression fragment. The length of the introgression fragments of 49 ILs from TX-1046 was 2,783.60, accounting for 71.60% of the whole introgression fragment^17^. Here, both the joint genetic linkage map and 115 ILs were employed to map QTLs of fiber quality traits.

### Measurement of fiber-related traits

In mid-October of 2013, Pop1 and Pop2 progenies were planted in Jiangpu Breeding Station of Nanjing Agricultural University (Nanjing, Jiangsu Province, China) (E1). The plots were 5 m long and 0.8 m apart, with a plant spacing of 40 cm and each line was planted in randomized block design for three replicates (totally 3 rows per IL). Fifteen bolls from each Pop2 individual were harvested, and 25 bolls from each Pop1 familyin the middle of each row were hand harvested from the internal middle parts of the plants (about 3 bolls per plant). In mid-October of 2014, as per the previous planting patterns, Pop1 and Pop2 families were planted in Jiangpu Breeding Station (E2) and Dafeng in Jiangsu Province (E3). Twenty-five bolls from each family in the middle of each row were hand harvested from the internal middle parts of the plants (about 3 bolls per plant). In mid-March of 2015, 25 bolls from each block in Pop1 and Pop2 family (about 3 bolls per plant) were hand harvested from Sanya in Hainan Province (E4). Fiber samples from all four environments were ginned by roller. The fiber qualities were evaluated by HVI: 2.5% fiber length (FL, mm), fiber strength (FS, cN/tex), micronaire (MIC), fiber elongation (FE, %) and fiber uniformity (FU, %). Basic statistical parameters, correlation coefficients and phenotypic variation were calculated for fiber-related traits of the 66 and 49 ILs from Pop1 and Pop2 using SPSS v18.0 (SPSS, Chicago, IL, USA), respectively.

### QTL mapping

A likelihood ratio test based on stepwise regression (RSTEP-LRT) was used to detect QTLs of non-idealized CSILs^32^,^40^. QTL IciMapping 3.0 (http://www.isbreeding.net) was used to detect the effects of QTL of non-idealized CSILs. An LOD (likelihood of odds) of threshold 2.5 was used to declare significant additive QTLs. The resulting linkage map and QTLs were drawn using MapChart ver.2.2 software^41^. The name of each QTL includes a “q” followed by an abbreviation of the trait name, the population type, the chromosome or linkage group and a serial number to distinguish different QTLs of the same trait on the same chromosome.

### Comparison of the QTLs and introgression segments

QTLs for fiber quality traits were detected in two distinct IL populations. One set of ILs was developed using *G. hirsutum* L. acc. TM-1 as the recipient parent and *G. hirsutum* race *richmondii* acc. TX-256 as the donor parent. We named this Pop1 to distinguish it from the other IL population, developed using *G. hirsutum* L. acc. TM-1 as the recipient parent and *G. hirsutum* race *yucatanense* acc. TX-1046 as the donor parent in this study (Pop2). All additive QTLs with an LOD threshold of 2.5 were selected for further analysis.

### DNA extraction, PCR amplification and electrophoresis

Total genomic DNA was extracted from young leaves using a modified CTAB method (Paterson *et al*. 1993). SSR-PCR amplifications were performed using a Peltier Thermal Cycler-225 (MJ Research)^42^.

## Additional Information

**How to cite this article**: Zhang, S.-W. *et al*. Mapping of fiber quality QTLs reveals useful variation and footprints of cotton domestication using introgression lines. *Sci. Rep.* **6**, 31954; doi: 10.1038/srep31954 (2016).

## Supplementary Material

Supplementary Information

## Figures and Tables

**Table 1:** Basic parameters of five fiber quality traits in four environments from two IL populations.

Traits	Pops	Environments	ILs							TM-1 Mean ± SD
			Min	Max	Mean	SD	CV(%)	Skew	Kurt	
FL	Pop1	E1	25.27	34.04	30.06	2.33	7.74	−0.07	−1.21	30.01 ± 0.42
		E2	26.92	34.22	30.48	1.45	4.75	−0.05	−0.29	31.36 ± 0.28
		E3	27.36	31.88	29.33	1.11	3.80	0.35	−0.59	29.97 ± 0.35
		E4	25.59	30.83	28.14	1.24	4.40	0.23	−0.51	29.11 ± 0.98
	Pop2	E1	23.20	33.05	29.30	2.61	8.89	−0.66	−0.73	30.01 ± 0.42
		E2	26.91	32.66	29.94	1.41	4.72	−0.52	−0.49	31.36 ± 0.28
		E3	24.93	32.66	28.74	1.48	5.14	−0.25	0.77	29.97 ± 0.35
		E4	23.88	29.72	27.03	1.23	4.55	−0.07	0.21	29.11 ± 0.98
FS	Pop1	E1	23.20	39.90	32.30	4.15	12.85	−0.23	−1.20	32.00 ± 0.53
		E2	28.45	38.37	33.02	3.20	9.69	0.33	−0.23	32.03 ± 0.24
		E3	27.77	34.40	30.42	2.60	8.55	0.32	0.15	29.63 ± 0.36
		E4	25.25	34.17	29.43	2.91	9.89	0.51	0.15	29.76 ± 0.97
	Pop2	E1	22.20	39.40	31.00	4.00	12.91	−0.22	−1.25	32.00 ± 0.53
		E2	28.66	37.81	32.88	2.71	8.24	0.22	−0.16	32.03 ± 0.24
		E3	27.47	34.10	30.07	2.63	8.75	0.75	0.88	29.63 ± 0.36
		E4	24.83	33.37	27.86	2.56	9.19	0.47	−0.30	29.76 ± 0.97
MIC	Pop1	E1	4.10	6.00	5.21	0.21	4.03	−0.27	−0.89	5.27 ± 0.12
		E2	3.68	5.63	4.81	0.19	3.95	−0.53	1.63	4.84 ± 0.08
		E3	3.47	5.27	4.51	0.17	3.77	−0.11	−0.05	4.57 ± 0.11
		E4	2.60	5.09	3.69	0.22	5.96	0.12	0.98	3.72 ± 0.44
	Pop2	E1	3.80	6.20	5.18	0.24	4.63	0.08	−0.34	5.27 ± 0.12
		E2	3.68	5.79	4.80	0.12	2.50	−0.21	0.73	4.84 ± 0.08
		E3	3.57	5.57	4.43	0.15	3.39	0.14	0.69	4.57 ± 0.11
		E4	2.26	4.72	3.52	0.19	5.40	−0.06	0.54	3.72 ± 0.44
FU	Pop1	E1	83.50	90.10	86.08	6.23	7.24	0.39	0.60	83.73 ± 1.06
		E2	83.57	87.47	85.87	5.76	6.71	−0.18	0.21	85.17 ± 0.70
		E3	82.20	85.50	84.00	4.68	5.57	−0.07	0.03	84.37 ± 0.89
		E4	81.93	86.53	84.30	3.26	3.87	−0.05	−1.21	85.17 ± 0.84
	Pop2	E1	84.10	91.50	86.43	6.53	7.56	0.93	1.14	83.73 ± 1.06
		E2	83.20	87.17	85.82	4.79	5.58	−0.52	0.88	85.17 ± 0.70
		E3	82.40	85.73	84.30	5.67	6.73	−0.21	0.70	84.37 ± 0.89
		E4	80.30	86.55	83.35	3.35	4.02	0.02	−0.26	85.17 ± 0.84
FE	Pop1	E1	3.60	7.10	5.05	0.41	8.03	0.26	−0.70	5.10 ± 0.19
		E2	7.77	11.97	9.53	0.80	8.35	0.67	0.40	9.20 ± 0.27
		E3	9.27	13.30	11.16	0.93	8.29	0.00	−0.75	11.67 ± 0.22
		E4	6.37	6.93	6.66	0.14	2.12	−0.32	0.34	6.80 ± 0.10
	Pop2	E1	3.70	9.83	5.71	0.36	6.35	0.96	1.17	5.10 ± 0.19
		E2	8.53	11.33	9.60	0.67	6.96	0.39	−0.43	9.20 ± 0.27
		E3	8.60	14.27	11.43	1.12	9.79	0.24	0.36	11.67 ± 0.22
		E4	6.30	7.13	6.57	0.15	2.29	0.88	2.90	6.80 ± 0.10

Pop1: the population of TM-1 × TX-256, Pop2: the population of TM-1 × TX-1046; E1: Nanjing, Jiangsu Province in 2013, E2: Nanjing, Jiangsu Province in 2014, E3: Dafeng, Jiangsu Province in 2014, E4: Sanya, Hainan Province in 2015.

**Table 2:** ANOVA for fiber quality traits in the two IL populations.

Traits and source	**Pop1**				**Pop2**			
	SS	df	MS	*F*	SS	df	MS	*F*
FL								
ILs(A)	1442.97	65	22.20	21.79**	1252.13	48	26.09	26.09**
Environment(B)	605.49	3	201.83	198.15**	689.82	3	229.94	229.94**
A × B	549.86	195	2.82	2.77**	568.00	144	3.94	3.94**
FS								
ILs(A)	3077.24	65	47.34	44.03**	1696.46	48	35.34	35.34**
Environment(B)	1494.87	3	498.29	463.47**	1865.58	3	621.86	621.86**
A × B	3180.89	195	16.31	15.17**	2833.42	144	19.68	19.68**
MIC								
ILs(A)	70.65	65	1.09	6.01**	78.42	48	1.63	11.60**
Environment(B)	232.13	3	77.38	427.89**	221.80	3	73.93	524.96**
A × B	61.35	195	0.31	1.74**	45.77	144	0.32	2.26**
FU								
ILs(A)	265.89	65	4.09	4.04**	298.49	48	6.22	6.22**
Environment(B)	648.96	3	216.32	213.48**	865.66	3	288.55	288.55**
A × B	559.32	195	2.87	2.83**	461.45	144	3.20	3.20**
FE								
ILs(A)	288.23	65	4.43	11.04**	249.96	48	5.21	12.96**
Environment(B)	4406.71	3	1468.90	3655.58**	3054.20	3	1018.07	2533.61**
A × B	204.61	195	1.05	2.61**	254.59	144	1.768	4.40**

*: stands for the significant level p = 0.05, **: stands for the significant level p = 0.01; Pop1: the population of TM-1 × TX-256, Pop2: the population of TM-1 × TX-1046; FL: Fiber length, FS: Fiber strength, FE: Fiber elongation, FU: Fiber uniformity, MIC: Micronaire.

**Table 3:** Correlation coefficients of the five traits in four environments from the two IL populations.

Populations	Environments	Traits	FL	FS	MIC	FU	FE
Pop1	E1	FL	1				
		FS	0.226*	1			
		MIC	−0.397**	−0.404**	1		
		FU	0.336**	0.400**	−0.109	1	
		FE	−0.736**	−0.554**	0.389**	−0.425**	1
	E2	FL	1				
		FS	0.471**	1			
		MIC	−0.510**	−0.238*	1		
		FU	0.038	0.357**	0.054	1	
		FE	−0.393**	−0.414**	0.357**	0.111	1
	E3	FL	1				
		FS	0.414**	1			
		MIC	−0.509**	−0.184*	1		
		FU	−0.098	0.179*	0.296*	1	
		FE	−0.528**	−0.314**	0.326**	0.18	1
	E4	FL	1				
		FS	0.692**	1			
		MIC	−0.237*	−0.193*	1		
		FU	0.734**	0.650**	−0.118	1	
		FE	0.394**	0.459**	0.201*	0.421**	1
Pop2	E1	FL	1				
		FS	0.326*	1			
		MIC	−0.500**	−0.345*	1		
		FU	0.467**	0.049	−0.359*	1	
		FE	−0.579**	−0.538**	0.291*	−0.278	1
	E2	FL	1				
		FS	0.329*	1			
		MIC	−0.508**	−0.301*	1		
		FU	0.066	0.278	0.119	1	
		FE	−0.683**	−0.484**	0.518**	−0.142	1
	E3	FL	1				
		FS	0.465**	1			
		MIC	−0.639**	−0.373**	1		
		FU	0.121	0.365**	−0.142	1	
		FE	−0.674**	−0.531**	0.456**	−0.265	1
	E4	FL	1				
		FS	0.849**	1			
		MIC	−0.312*	−0.297*	1		
		FU	0.774**	0.773**	−0.144	1	
		FE	0.364**	0.471**	0.280*	0.346*	1

*: stands for the significant level p = 0.05, **: stands for the significant level p = 0.01; Pop1: the population of TM-1 × TX-256, Pop2: the population of TM-1 × TX-1046; E1: Nanjing, Jiangsu Province in 2013, E2: Nanjing, Jiangsu Province in 2014, E3: Dafeng, Jiangsu Province in 2014, E4: Sanya, Hainan Province in 2015; FL: Fiber length, FS: Fiber strength, FE: Fiber elongation, FU: Fiber uniformity, MIC: Micronaire.

**Table 4:** Results of QTL mapping for fiber quality traits in four environments.

Traits	Pops	QTLs	Markers	Chrs.	Position	LOD				PVE(%)				Additive effect			
						E1	E2	E3	E4	E1	E2	E3	E4	E1	E2	E3	E4
FE	Pop1	qFE-Pop1-A11-1	HAU1809	A11	0	2.92		2.86		6.19		5.85		−0.26		−0.26	
		qFE-Pop1-A3-1	NAU429	A3	41.85	2.89	4.65	3.04		6.03	16.85	7.03		0.39	0.64	0.43	
		qFE-Pop1-A7-1	NAU7446	A7	59.66	3.24		3.19	3.44	8.68		8.32	9.53	−0.48		−0.44	−0.77
		qFE-Pop1-A8-1	BNL3255c	A8	26.33	2.74	2.65	3.24		5.00	4.78	8.31		−0.38	−0.37	−0.50	
		qFE-Pop1-D13-1	cgr5446	D13	70.35	2.6		2.88		4.11		5.95		−0.32		−0.39	
		qFE-Pop1-D5-1	NAU455	D5	63.79	2.97	2.74	2.81		6.51	4.20	5.48		−0.35	−0.28	−0.33	
	Pop2	qFE-Pop2-A13-1	TMB04	A13	0	3.79			2.80	15.51			7.27	1.87			1.04
		qFE-Pop2-A5-1	dPL0641	A5-2	21.45	2.65	2.62			5.91	5.68			1.15	0.55		
		qFE-Pop2-A7-2	NAU1085	A7	56.56	2.74	3.09	3.26		6.81	10.11	11.58		−0.42	−0.25	−0.44	
		qFE-Pop2-A9-1	NAU3358	A9	131.99		2.57	3.65			5.18	14.33			−0.52	−1.46	
		qFE-Pop2-D8-1	TML21	D8	64.86	3.00			3.96	8.99			11.85	−0.50			−0.16
FL	Pop1	qFL-Pop1-A11-1	HAU1809	A11	0	3.04		2.83		7.99		5.61		−0.70		−0.30	
		qFL-Pop1-A2-1	cot064a	A2	46.56	3.42			3.03	9.43			7.95	−1.81			−1.31
		qFL-Pop1-A8-1	BNL3255c	A8	26.33	3.41	2.73	2.53	2.91	8.83	4.97	4.11	4.87	0.73	0.61	0.37	0.35
		qFL-Pop1-D1-1	JESPR243	D1	73.07			3.22	2.65			8.17	4.44			1.29	1.05
		qFL-Pop1-D13-1	cgr5446	D13	70.35	3.02		2.62		7.89		4.23		1.05		0.39	
		qFL-Pop1-D2-1	NAU2312a	D2	73.80	3.24	3.29	2.57	2.61	8.77	8.99	4.90	4.63	−1.22	−0.83	−0.89	−0.40
		qFL-Pop1-D5-1	NAU455	D5	63.79	2.63		2.94	3.09	4.31		6.32	7.35	0.73		0.42	0.51
		qFL-Pop1-D6-1	NAU2714	D6	71.81			3.72	3.92			11.31	12.54			1.08	1.26
		qFL-Pop1-D8-1	cgr5537	D8	25.50			2.59	2.69			4.03	4.68			−0.26	−0.31
		qFL-Pop1-D8-2	cgr6736	D8	64.40		2.72	2.51			4.92	3.47			−0.23	−0.30	
	Pop2	qFL-Pop2-A3-1	NAU1167a	A3	86.77	2.52		2.92	2.51	3.09		5.85	3.41	1.33		0.70	0.51
		qFL-Pop2-A5-1	dPL0641	A5-2	21.45	2.87	2.56	3.37	2.52	4.86	4.03	10.04	3.78	−2.58	−1.00	−1.79	−0.95
		qFL-Pop2-A7-1	NAU1085	A7	56.56	2.85	4.48	2.68	2.56	4.08	15.53	4.36	4.06	−0.82	−0.76	−0.44	−0.33
		qFL-Pop2-A8-2	cot065	A8	22.80		2.93				5.97				0.33		
		qFL-Pop2-A8-3	NAU2293b	A8	93.63	3.29			2.56	8.40			4.09	−3.11			−0.98
		qFL-Pop2-A9-1	NAU3358	A9	131.99		2.83	3.76			5.87	11.27			1.21	2.02	
		qFL-Pop2-D1-2	NAU3254	D1	96.96	2.53		2.86		3.81		5.11		−0.44		−0.16	
		qFL-Pop2-D11-2	BNL1705	D11	11.58		2.54	2.98	2.82		4.04	5.87	4.70		−0.51	−0.70	−0.57
		qFL-Pop2-D11-1	NAU6224	D11	113.70	2.85	3.37		3.55	4.45	9.45		10.59	−1.94	−1.53		−1.60
		qFL-Pop2-D2-2	HAU2511	D2	70.94	2.53		2.67	2.94	3.24		4.12	4.72	−0.37		−0.47	−0.26
		qFL-Pop2-D8-3	TML21	D8	64.86	3.02	3.06	2.62	2.98	7.13	7.41	3.67	4.97	−0.98	−0.48	−0.43	−0.33
FS	Pop1	qFS-Pop1-A13-1	dc30178	A13	57.23	2.51	2.85	2.71	2.72	2.03	3.12	4.83	4.91	−3.53	−1.46	−1.17	−1.69
		qFS-Pop1-A3−1	NAU5233	A3	88.45	2.94	2.52		2.51	4.33	2.65		3.49	−0.77	−0.27		−0.41
		qFS-Pop1-A5-1	NAU1223	A5-1	0	3.08	3.27	4.05	2.7	6.23	5.46	13.34	4.77	1.35	0.69	0.51	0.44
		qFS-Pop1-A6-1	dPL0617c	A6	79.46		3.38	4.24	3.47		7.19	14.47	9.75		2.72	2.02	2.39
		qFS-Pop1-A8-1	BNL3255c	A8	26.33		2.53	2.55	2.54		2.85	3.80	2.70		0.62	0.22	0.35
		qFS-Pop1-D2-1	NAU2312a	D2	73.80	2.97	2.79	2.84	2.53	4.57	3.1	5.80	2.30	−2.71	−1.00	−0.40	−0.42
		qFS-Pop1-D8-1	cgr5537	D8	25.50		2.98	3.90			4.63	12.43			0.67	0.54	
	Pop2	qFS-Pop2-A13-2	BNL2449	A13	56.29	2.83	2.59		2.51	3.85	3.85		3.08	−3.11	−1.61		−0.75
		qFS-Pop2-A8-2	cot065	A8	22.80		2.53	2.65			3.49	2.93			0.45	0.54	
		qFS-Pop2-D2-2	cgr5033	D2	26.34		2.54	2.94			3.74	3.10			2.55	2.09	
		qFS-Pop2-D2-3	HAU2511	D2	70.94		2.91	2.65	2.88		5.78	2.95	4.90		−0.34	−0.26	−0.74
		qFS-Pop2-D5-1	dPL0137	D5	111.55	2.52			3.68	3.79			6.64	3.47			2.21
MIC	Pop1	qMIC-Pop1-A2-1	NAU1529	A2	100.69	3.13	3.79	3.00	4.48	6.59	9.57	6.17	14.89	0.18	0.41	0.39	0.70
		qMIC-Pop1-A3-1	NAU429	A3	41.85		3.19	4.03			6.48	13.23			−0.14	−0.23	
		qMIC-Pop1-D1-1	JESPR243	D1	73.07	2.93	4.91	3.91		5.27	15.37	8.50		−0.51	−0.57	−0.53	
		qMIC-Pop1-D1-2	NAU3254	D1	96.96		3.41	3.13			6.64	7.55			−0.49	−0.17	
		qMIC-Pop1-D11-1	cgr5578b	D11	6.75		2.55	2.54	2.85		4.28	4.31	4.99		0.71	0.83	0.12
		qMIC-Pop1-D8-1	cgr6736	D8	64.40	2.68	2.51		2.74	4.63	4.28		4.76	−0.16	−0.25		−0.63
	Pop2	qMIC-Pop2-A6-1	BNL2569	A6	40.69	3.55	2.60	3.57	3.89	8.56	3.53	8.68	8.25	−0.70	−0.35	−0.55	−0.65
		qMIC-Pop2-A8-1	cot065	A8	22.80	2.53	2.65	2.75	2.54	3.14	3.90	4.82	3.25	0.13	0.14	0.15	0.11
		qMIC-Pop2-D1-3	NAU3254	D1	96.96		2.52		2.75		3.31		3.63		−0.61		−0.59
		qMIC-Pop2-D11-2	BNL1705	D11	11.58				3.64				7.27				0.61
		qMIC-Pop2-D2-1	HAU2511	D2	70.94	2.52	2.51	2.51		3.07	3.23	3.98		−0.92	−0.60	−0.54	
		qMIC-Pop2-D8-2	TML21	D8	64.86	2.54		2.51	2.87	3.27		3.92	3.75	−0.12		−0.60	−0.75
FU	Pop1	qFU-Pop1-A2-1	cot064a	A2	46.56	3.19	2.65		2.83	6.62	4.47		5.64	−1.29	−0.66		−1.21
		qFU-Pop1-D6-1	JESPR302	D6	32.60		4.18				14.12				−1.17		
	Pop2	qFU-Pop2-A5-1	cgr6708a	A5-1	9.33			3.05	2.55			9.35	3.5			−0.73	1.07
		qFU-Pop2-A7-1	NAU1085	A7	56.56			2.81	2.52			7.66	3.39			−0.22	−0.35

Pop1: the population of TM-1 × TX-256, Pop2: the population of TM-1 × TX-1046; E1: Nanjing, Jiangsu Province in 2013, E2: Nanjing, Jiangsu Province in 2014, E3: Dafeng, Jiangsu Province in 2014, E4: Sanya, Hainan Province in 2015; FL: Fiber length, FS: Fiber strength, FE: Fiber elongation, FU: Fiber uniformity, MIC: Micronaire.

**Table 5:** Common QTLs for five traits detected using the two ILs populations.

Chr.	Name of QTLs	QTLs detected in introgression segment(Additive effect)	Markers associated with QTLs	QTL position (cM)	Chr.	Name of QTLs	QTLs detected in introgression segment(Additive effect)	Markers associated with QTLs	QTL position (cM)
**Pop1**					**Pop2**				
A7	qFE-Pop1-A7-1	FE(−)	NAU7446	59.66	A7	qFE-Pop2-A7-2	FE(−)	NAU1085	56.56
A8	qFL-Pop1-A8-1	FL(+)	BNL3255c	26.33	A8	qFL-Pop2-A8-2	FL(+)	cot065	22.80
D2	qFL-Pop1-D2-1	FL(−)	NAU2312a	73.80	D2	qFL-Pop2-D2-2	FL(−)	HAU2511	70.94
D8	qFL-Pop1-D8-2	FL(−)	cgr6736	64.40	D8	qFL-Pop2-D8-3	FL(−)	TML21	64.86
A13	qFS-Pop1-A13-1	FS(−)	dc30178	57.23	A13	qFS-Pop2-A13-2	FS(−)	BNL2449	56.29
A8	qFS-Pop1-A8-1	FS(+)	BNL3255c	26.33	A8	qFS-Pop2-A8-2	FS(+)	cot065	22.80
D2	qFS-Pop1-D2-1	FS(−)	NAU2312a	73.80	D2	qFS-Pop2-D2-3	FS(−)	HAU2511	70.94
D1	qMIC-Pop1-D1-2	MIC(+)	NAU3254	96.96	D1	qMIC-Pop2-D1-3	MIC(+)	NAU3254	96.96
D8	qMIC-Pop1-D8-1	MIC(+)	cgr6736	64.40	D8	qMIC-Pop2-D8-2	MIC(+)	TML21	64.86

Pop1: the population of TM-1 × TX-256, Pop2: the population of TM-1 × TX-1046; FL: Fiber length, FS: Fiber strength, FE: Fiber elongation, FU: Fiber uniformity, MIC: Micronaire. (+) means desirable QTL, (−) means undesirable QTL.

**Table 6:** Distribution of QTL clusters.

Cluster name	Approximate position on chromosome (cM)	Number of QTLs	Name of QTLs in Pop1	Name of QTLs in Pop2	Compared with clusters of Said *et al*. (2015)^13^
A7-cluster	56.56–59.66	4	qFE-Pop1-A7-1(−)	qFE-Pop2-A7-2(−)	c7-cluster-Gh×Gb-4:55-79cM
				qFL-Pop2-A7-1(−)	
				qFU-Pop2-A7-1(−)	
A8-cluster	22.80–26.33	6	qFL-Pop1-A8-1(+)	qFL-Pop2-A8-2(+)	c8-cluster-Gh-2:21-31cM
			qFS-Pop1-A8-1(+)	qFS-Pop2-A8-2(+)	
			qFE-Pop1-A8-1(−)	qMIC-Pop2-A8-1(−)	
D1-cluster	73.07–96.96	5	qFL-Pop1-D1-1(+)	qFL-Pop2-D1-2(−)	c15-cluster-Gh-3:49-68cM
			qMIC-Pop1-D1-1(+)	qMIC-Pop2-D1-3(+)	
			qMIC-Pop1-D1-2(+)		
D2-cluster	70.94–73.80	5	qFL-Pop1-D2-1(−)	qFL-Pop2-D2-2(−)	c14-cluster-Gh-2:76-91cM
			qFS-Pop1-D2-1(−)	qFS-Pop2-D2-3(−)	
				qMIC-Pop2-D2-1(+)	
D8-cluster	64.40–64.86	5	qFL-Pop1-D8-2(−)	qFL-Pop2-D8-3(−)	c24-cluster-Gh-2:41-62cM
			qMIC-Pop1-D8-1(+)	qMIC-Pop2-D8-2(+)	
				qFE-Pop2-D8-1(−)	

Pop1: the population of TM-1 × TX-256, Pop2: the population of TM-1 × TX-1046; FL: Fiber length, FS: Fiber strength, FE: Fiber elongation, FU: Fiber uniformity, MIC: Micronaire. (+) means desirable QTL, (−) means undesirable QTL.

**Table 7:** Comparison of hotspots and QTLs from previous reports with this study.

QTLs	Markers	Chrs.	Position	QTLs and hotspots from previous reports	Previous reports
qFE-Pop2-A13-1(+)	TMB04	A13	0	qFE-F_2:3_-A13-1	Zhang *et al*. (2015)^16^
qFE-Pop2-A5-1(+)	dPL0641	A5-2	21.45	c5-mQTL-FE-Gh-1:0-20 cM	Said *et al*. (2015)^13^
qFE-Pop2-A7-2(−)	NAU1085	A7	56.56	qFE-C7-1	Wang *et al*. (2013)^14^
qFE-Pop1-A7-01(−)	NAU7446	A7	59.66	qFE-C7-1	Wang *et al*. (2013)^14^
qFE-Pop2-A9-1(−)	NAU3358	A9	131.99	qFE9-1	Yang *et al*. (2015)^37^
qFE-Pop1-D5-1(−)	NAU455	D5	63.79	c19-mQTL-FE-Gh × Gb-2:32-52 cM	Said *et al*. (2015)^13^
qFL-Pop1-A2-1(−)	cot064a	A2	46.56	qFL02.1	Tan *et al*. (2015)^43^
qFL-Pop2-A3-1(+)	NAU1167a	A3	86.77	qFL-F_2:3_-A3-1	Zhang *et al*. (2015)^16^
qFL-Pop2-A5-1(−)	dPL0641	A5-2	21.45	qFL-Chr5-1	Shang *et al*. (2015)^44^
qFL-Pop2-A7-1(−)	NAU1085	A7	56.56	qFL-C7-1	Wang *et al*. (2013)^14^
qFL-Pop2-A8-2(+)	cot065	A8	22.80	c8-mQTL-FL-Gh-3:0-32 cM	Said *et al*. (2015)^13^
qFL-Pop1-A8-1(+)	BNL3255c	A8	26.33	c8-mQTL-FL-Gh-3:0-32 cM	Said *et al*. (2015)^13^
qFL-Pop2-D11-2(−)	BNL1705	D11	11.58	c21-mQTL-FL-Gh-7:6-26 cM	Said *et al*. (2015)^13^
qFL-Pop2-D11-1(−)	NAU6224	D11	113.70	qFL-Chr21-1	Shang *et al*. (2015)^44^
qFL-Pop1-D5-1(+)	NAU455	D5	63.79	c19-mQTL-FL-Gh × Gb-6:40-60 cM	Said *et al*. (2015)^13^
qFL-Pop1-D8-1(−)	cgr5537	D8	25.50	c24-mQTL-FL-Gh-8:0-20 cM	Said *et al*. (2015)^13^
qFL-Pop1-D8-2(−)	cgr6736	D8	64.40	c24-mQTL-FL-Gh × Gb-7:49-70 cM	Said *et al*. (2015)^13^
qFL-Pop2-D8-3(−)	TML21	D8	64.86	c24-mQTL-FL-Gh × Gb-7:49-70 cM	Said *et al*. (2015)^13^
qFS-Pop1-A5-1(+)	NAU1223	A5-1	0	qFS5-1	Yang *et al*. (2015)^37^
qFS-Pop2-A8-2(+)	cot065	A8	22.80	c8-mQTL-FS-Gh-2:0-20 cM	Said *et al*. (2015)^13^
qFS-Pop1-A8-1(+)	BNL3255c	A8	26.33	c8-mQTL-FS-Gh-2:0-20 cM	Said *et al*. (2015)^13^
qFS-Pop2-D5-1(+)	dPL0137	D5	111.55	qFS-F_2_-D5-1	Zhang *et al*. (2015)^16^
qFS-Pop1-D8-1(+)	cgr5537	D8	25.50	c24-mQTL-FS-Gh-6:0-20 cM	Said *et al*. (2015)^13^
qFU-Pop1-A2-1(−)	cot064a	A2	46.56	qFU-C2-1	Wang *et al*. (2013)^14^
qMIC-Pop2-A6-1(+)	BNL2569	A6	40.69	qFM06.1	Tan *et al*. (2015)^43^
qMIC-Pop1-D11-1(−)	cgr5578b	D11	6.75	New	
qMIC-Pop2-D11-2(−)	BNL1705	D11	11.58	New	
qMIC-Pop1-D8-1(+)	cgr6736	D8	64.40	qFM24-1	Yang *et al*. (2015)^37^
qMIC-Pop2-D8-2(+)	TML21	D8	64.86	qFM24-2	Yang *et al*. (2015)^37^

Pop1: the population of TM-1 × TX-256, Pop2: the population of TM-1 × TX-1046; FL: Fiber length, FS: Fiber strength, FE: Fiber elongation, MIC: Micronaire. (+) means desirable QTL, (−) means undesirable QTL.
