# Supplementary material for: Mapping of fiber quality QTLs reveals useful variation and footprints of cotton domestication using introgression lines
Source: Sci Rep. 2016 Aug 23;6:31954. doi: 10.1038/srep31954 (PMC4994025; doi:10.1038/srep31954)
Supplement: Supplementary Information [file srep31954-s1.pdf]

Mapping of fiber quality QTLs reveals useful variation and footprints of cotton domestication using introgression lines

Shu-Wen Zhang[1], Xie-Fei Zhu[1], Liu-Chun Feng[1], Xiang Gao[1], Biao Yang[1], Tian-Zhen Zhang[1], and Bao-Liang Zhou[1]*

Legends

Fig. S1 The location and additive effect of QTLs for five traits detected in four environments.

Pop1: the population of TM-1×TX-256, Pop2: the population of TM-1×TX-1046; FL: Fiber length, FS: Fiber strength, FE: Fiber elongation, FU: Fiber uniformity, MIC: Micronaire. (+) means desirable QTL, (-) means undesirable QTL.

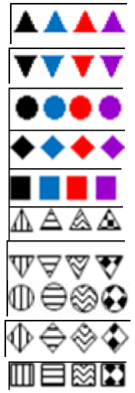

indicated QTLs for FE in E1, E2, E3 and E4 from Pop1, respectively;
indicated QTLs for FU in E1, E2, E3 and E4 from Pop1, respectively;
indicated QTLs for MIC in E1, E2, E3 and E4 from Pop1, respectively;
indicated QTLs for FL in E1, E2, E3 and E4 from Pop1, respectively;
indicated QTLs for FS in E1, E2, E3 and E4 from Pop1, respectively;
indicated QTLs for FE in E1, E2, E3 and E4 from Pop2, respectively;
indicated QTLs for FU in E1, E2, E3 and E4 from Pop2, respectively;
indicated QTLs for MIC in E1, E2, E3 and E4 from Pop2, respectively;
indicated QTLs for FL in E1, E2, E3 and E4 from Pop2, respectively;
indicated QTLs for FS in E1, E2, E3 and E4 from Pop2, respectively.

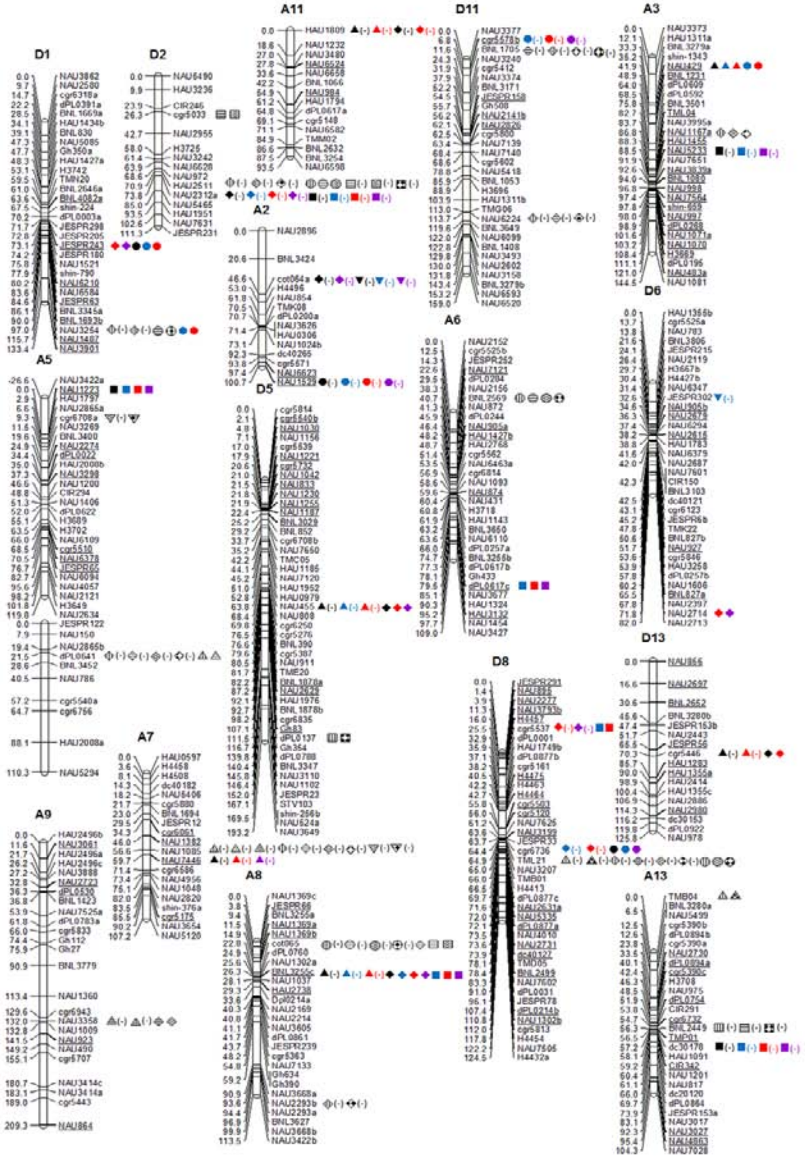
D1
D2
A11
D11
A3
A2
A5
A6
D6
D5
D13
A7
A9
D8
A8
A13

Table S1 Results of QTL mapping for yield-related traits in four environments

Traits	Pops	QTLs	Markers	Chrs.	Position	LOD				PVE(%)				Additive effect			
						E1	E2	E3	E4	E1	E2	E3	E4	E1	E2	E3	E4
BW	Pop1	qBW-Pop1-A12-1	dPL0531	A12	42.80		3.26	2.63			6.45	2.25			-0.62	-0.31	
		qBW-Pop1-A13-2	dPL0864	A13	69.70			3.06	2.83			5.14	3.60			-0.34	-0.42
		qBW-Pop1-A3-1	NAU429	A3	41.85		3.19		3.01		5.99		4.82		-0.26		-0.29
		qBW-Pop1-A3-2	NAU5233	A3	88.45			2.73	2.56			2.98	1.76			-0.10	-0.12
		qBW-Pop1-A3-3	NAU1071a	A3	101.61		3.70	2.68	2.84		9.32	2.61	3.67		0.26	0.11	0.20
		qBW-Pop1-A5-1	NAU1223	A5	0		2.57	2.51	2.51		1.87	1.49	1.47		-0.88	-0.17	-0.10
		qBW-Pop1-A6-1	dPL0617c	A6	79.46		2.92	2.69	2.67		4.21	2.66	2.54		-0.50	-0.34	-0.50
		qBW-Pop1-A8-1	NAU1369c	A8	0		2.63	2.92	2.58		2.27	4.22	1.93		-0.17	-0.20	-0.20
		qBW-Pop1-A9-1	dPL0530	A9	36.25			2.57	2.66			1.86	2.47			0.28	0.49
		qBW-Pop1-D1-1	dPL0003a	D1	70.21		2.76	2.76			3.19	3.18			-0.44	-0.37	
		qBW-Pop1-D10-1	HAU2200	D10	20.08		2.98	3.64			4.63	8.94			-0.53	-0.62	
		qBW-Pop1-D2-2	HAU3236	D2	9.87	2.67	2.94	2.67	2.97	1.94	4.37	2.52	4.54	-1.17	-0.20	-0.13	-0.26
		qBW-Pop1-D6-1	JESPR302	D6	32.60		2.51	3.06			1.44	5.16			0.29	0.47	
		qBW-Pop1-D8-1	cgr6736	D8	64.40	2.52	2.81	2.94	3.04	1.17	3.49	4.36	5.00	1.79	0.16	0.15	0.25
	Pop2	qBW-Pop2-A1-1	dc40052	A1	34.65		3.35	2.98	2.81		9.42	6.21	4.66		-0.27	-0.19	-0.20
		qBW-Pop2-A13-1	TMB04	A13	0		2.51	2.56	2.56		1.95	2.42	2.42		-0.32	-0.31	-0.37
		qBW-Pop2-D2-1	NAU5490	D2	0		2.65		2.69		3.19		3.57		-0.15		-0.16
		qBW-Pop2-D5-1	dPL0137	D5-1	111.55		3.85		2.68		13.60		3.51		0.85		0.45
		qBW-Pop2-D5-2	Gh354	D5-1	116.74		3.61	2.74	3.29		11.58	4.02	8.85		0.78	0.39	0.71
		qBW-Pop2-D7-1	dc40065	D7	79.81		2.55	2.59			2.32	2.58			-0.11	-0.10	
		qBW-Pop2-D7-2	NAU2657	D7	95.05		2.67	3.35	2.64		3.43	9.36	3.19		-0.43	-0.60	-0.43
		qBW-Pop2-D8-2	TML21	D8	64.86		2.98	2.58	2.65		6.21	2.62	3.25		0.20	0.11	0.15
		qBW-Pop2-D9-1	NAU462	D9	16.53		2.61	3.00			2.83	6.37			-0.28	-0.35	
LP	Pop1	qLP-Pop1-A11-1	HAU1809	A11	0	2.61	3.12	2.71	2.65	2.13	5.57	2.82	2.41	-0.52	-0.66	-0.43	-0.39
		qLP-Pop1-A13-2	cgr5390a	A13	23.78	4.55	4.39	3.97	4.19	14.52	13.57	11.02	12.36	2.26	1.70	1.40	1.45
		qLP-Pop1-A13-4	dPL0864	A13	69.70		2.80		2.61		3.43		2.13		-1.32		-0.93
		qLP-Pop1-A3-1	NAU429	A3	41.85	3.26		2.66	2.68	6.49		2.50	2.62	-1.39		-0.61	-0.62
		qLP-Pop1-A3-2	NAU5233	A3	88.45	2.73		2.89	4.03	2.99		4.05	11.38	-0.63		-0.52	-0.86

		qLP-Pop1-A3-3	NAU1071a	A3	101.61	3.51	2.96	3.05	3.01	8.11	4.52	5.11	4.86	1.25	0.72	0.70	0.67
		qLP-Pop1-A5-1	NAU2274	A5	24.93	2.61	2.74	2.52	2.63	2.16	3.01	1.55	2.26	-1.11	-1.02	-0.67	-0.79
		qLP-Pop1-A9-1	dPL0530	A9	36.25		2.69	2.61	2.65		2.70	2.13	2.44		-1.64	-1.33	-1.40
		qLP-Pop1-A9-2	dPL0783a	A9	61.77	2.51	2.54	2.79	2.51	1.43	1.65	3.36	1.44	-1.54	-1.28	-1.67	-1.08
		qLP-Pop1-A9-3	BNL3779	A9	90.92	2.82		2.55		3.55		1.74		-2.42		-1.21	
		qLP-Pop1-A9-4	TMN20	D1	59.55	2.78	2.56	2.64	2.56	3.33	1.80	2.32	1.80	-2.35	-1.34	-1.39	-1.20
		qLP-Pop1-D1-1	JESPR243	D1	73.07		2.56	2.71	2.55		1.83	2.81	1.73		-1.35	-1.53	-1.18
		qLP-Pop1-D2-2	HAU3236	D2	9.87			2.60	2.61			2.08	2.14			-0.52	-0.52
		qLP-Pop1-D2-3	NAU2312a	D2	73.80	2.69			2.56	2.67			1.80	2.10			1.20
		qLP-Pop1-D5-1	TME20	D5-1	81.66	2.59		2.51	2.64	2.01		1.47	2.32	-1.82		-1.11	-1.37
		qLP-Pop1-D5-2	NAU2503	D5-2	0	2.52	2.89		2.94	1.52	4.06		4.39	-1.59	-2.01		-1.88
		qLP-Pop1-D6-1	JESPR302	D6	32.60	2.51	3.04	2.71	2.65	1.47	5.03	2.83	2.39	1.56	2.24	1.54	1.39
	Pop2	qLP-Pop2-A10-1	dc40188	A10	33.52	5.03			2.77	2.93			4.29	-1.60			-2.13
		qLP-Pop2-A12-1	STV033	A12	32.56		2.59	2.98	2.52		2.67	6.22	2.05		-1.76	-2.35	-1.47
		qLP-Pop2-A13-1	TMB04	A13	0		2.65	2.56			3.24	2.46			-1.94	-1.47	
		qLP-Pop2-A13-3	BNL2449	A13	56.29		3.23	2.63	2.81		8.39	3.07	4.66		-3.12	-1.65	-2.22
		qLP-Pop2-A6-1	BNL2569	A6	40.69		2.66	2.99	3.38		3.37	6.27	9.64		-1.97	-2.36	-3.19
		qLP-Pop2-A6-2	NAU3677	A6	85.10			2.70	2.70			3.70	3.70			-1.81	-1.98
		qLP-Pop2-A8-1	NAU3605	A8	41.10		2.64	2.54			3.14	2.22			-1.91	-1.40	
		qLP-Pop2-D11-1	BNL1705	D11	11.58		3.02	2.88	3.10		6.54	5.27	7.25		2.75	2.16	2.77
		qLP-Pop2-D2-1	NAU5490	D2	0		2.97	3.29	2.84		6.10	8.85	4.94		0.97	1.02	0.83
		qLP-Pop2-D7-1	NAU2657	D7	95.05		2.51		2.64		1.94		3.15		-1.50		-1.82
		qLP-Pop2-D9-1	NAU462	D9	16.53			2.91	2.76			5.53	4.20			-1.58	-1.50
		qLP-Pop2-D9-2	BNL3383	D9	64.52		3.90	3.27	2.52		13.96	8.67	2.02		-4.02	-2.77	-1.46
SI	Pop1	qSI-Pop1-A11-1	HAU1809	A11	0		2.53		3.03		1.61		4.98		-0.15		-0.26
		qSI-Pop1-A12-1	dPL0531	A12	42.80	2.96		2.54	2.68	3.71		1.69	2.60	-1.15		-0.57	-0.68
		qSI-Pop1-A13-1	cgr5390a	A13	23.78	5.03	3.84	3.68	3.19	17.36	10.21	9.16	5.99	1.14	0.64	0.61	0.47
		qSI-Pop1-A3-1	NAU1071a	A3	101.61	2.84			2.54	3.05			1.67	0.35			0.18
		qSI-Pop1-A5-1	NAU2274	A5	24.93	2.54		2.74		1.35		3.04		-0.41		-0.45	
		qSI-Pop1-A6-2	dPL0617c	A6	79.46	3.11	2.56	2.64		4.55	1.81	2.37		-1.27	-0.59	-0.67	
		qSI-Pop1-A8-1	NAU1369c	A8	0	2.56	3.45		2.63	1.51	7.72		2.28	-0.34	-0.56		-0.29
		qSI-Pop1-A9-1	dPL0530	A9	36.25			2.87	3.91			3.93	10.64			0.87	1.37
		qSI-Pop1-A9-2	dPL0783a	A9	61.77	4.04	2.50	2.56		9.44	1.42	1.82		1.83	0.52	0.59	
		qSI-Pop1-D1-1	dPL0003a	D1	70.21	3.05	3.40	2.93	2.52	4.19	7.39	4.27	1.50	-1.22	-1.18	-0.90	-0.51

	qSI-Pop1-D10-1	HAU2200	D10	20.08	3.55	3.82	2.67	2.59	6.91	10.07	2.54	2.01	-1.56	-1.38	-0.70	-0.59
	qSI-Pop1-D2-2	HAU3236	D2	9.87	2.54	3.75	3.69	3.53	1.36	9.62	9.26	8.20	0.27	0.54	0.53	0.48
	qSI-Pop1-D6-1	NAU2714	D6	71.81	3.44	3.23	2.82		6.32	6.29	3.56		1.06	0.78	0.59	
	qSI-Pop1-D7-1	HAU2662	D7	33.02	3.01	2.76	2.51		4.02	3.16	1.48		-1.19	-0.77	-0.53	
	qSI-Pop1-D8-1	cgr6736	D8	64.40		2.94	2.72			4.40	2.89			0.32	0.26	
Pop2	qSI-Pop2-A10-1	dc40188	A10	33.52	5.60	2.65		2.63	3.98	3.20		3.09	-1.72	-0.82		-0.75
	qSI-Pop2-A6-1	cgr6814	A6	56.85		2.59	2.55	3.01		2.68	2.33	6.48		0.75	0.65	1.08
	qSI-Pop2-A6-3	NAU3677	A6	85.10		2.54	3.43			2.25	10.10			0.69	1.35	
	qSI-Pop2-D2-1	NAU5490	D2	0		2.87	2.88	2.64		5.21	5.31	3.17		-0.38	-0.36	-0.28
	qSI-Pop2-D5-1	dPL0137	D5-1	111.55		3.87	2.61	2.89		13.75	2.90	5.35		1.69	0.72	0.98
	qSI-Pop2-D5-2	Gh354	D5-1	116.74		2.71		2.74		3.82		4.07		0.89		0.86
	qSI-Pop2-D8-2	TML21	D8	64.86		2.89		3.74		5.38		12.62		0.37		0.53
